# Optimization Strategy of 4H-SiC Separated Absorption Charge and Multiplication Avalanche Photodiode Structure for High Ultraviolet Detection Efficiency

**DOI:** 10.1186/s11671-019-3227-0

**Published:** 2019-12-30

**Authors:** Jianquan Kou, KangKai Tian, Chunshuang Chu, Yonghui Zhang, Xingye Zhou, Zhihong Feng, Zi-Hui Zhang

**Affiliations:** 1 State Key Laboratory of Reliability and Intelligence of Electrical Equipment, Hebei University of Technology, 5340 Xiping Road, Beichen District, Tianjin, 300401 People’s Republic of China; 2 School of Electronics and Information Engineering, Hebei University of Technology, Key Laboratory of Electronic Materials and Devices of Tianjin, 5340 Xiping Road, Beichen District, Tianjin, 300401 People’s Republic of China; 3 National Key Laboratory of ASIC, Hebei Semiconductor Research Institute, Shijiazhuang, 050051 People’s Republic of China

**Keywords:** 4H-SiC, Separated absorption charge and multiplication (SACM), Avalanche photodiode (APD), Dark current, Breakdown voltage, Responsivity

## Abstract

In this work, parametric investigations on structural optimization are systematically made for 4H-SiC-based separated absorption charge and multiplication (SACM) avalanche ultraviolet photodiode (UV APD). According to our results, the breakdown voltage can be strongly affected by the thickness for the multiplication layer and the doping concentration for the charge control layer. The thickness for the n-type ohmic contact layer, the absorption layer, and the charge control layer can remarkably affect the light penetration depth, which correspondingly influences the number of photo-generated electron-hole pairs, and therefore the aforementioned layer thickness has a strong impact on the responsivity for SACM APD. For enhancing the responsivity of the APD, we require a reduced energy band barrier height at the interface of the optical absorption layer and the charge control layer, so that the promoted carrier transport into the multiplication layer can be favored. In addition, we investigate positive beveled mesas with smaller angles so as to reduce the electric field at the mesa edge. Thus, the dark current is correspondingly suppressed.

## Introduction

As a wide bandgap semiconductor material, silicon carbide (SiC) and aluminum gallium nitride (AlGaN) exhibit excellent material characteristics such as high critical electric field, better anti-radiation effect, and good thermal conductivity, which make it suitable for ultraviolet (UV) detection [1–3]. The adjustable bandgap between 3.4 and 6.2 eV for the AlGaN-based photodetectors enables the controllable cutoff response wavelength ranging from 365 to 200 nm. However, due to the difficulty in growing high-quality Al-rich AlGaN compounds, the dark current for the AlGaN-based photodetectors is higher than that of SiC-based counterparts [4]. Therefore, SiC-based photodetectors have gained extensive research interest. Up to date, 4H-SiC-based solid-state ultraviolet detectors comprise Schottky barrier diode, metal-semiconductor-metal (MSM) photodiode, p-i-n photodiode, and avalanche photodiode (APD) [5–9]. Due to the high avalanche gain, the small dark current and the low noise, 4H-SiC ultraviolet APDs have great application prospects in many cutting-edge fields that require weak ultraviolet signal detection, such as fire warning, quantum communication, and missile detection [10–12]. However, SiC material has a low light absorption coefficient, thus a common photodiode structure with a thin multiplication layer is difficult to achieve high quantum efficiency. The problem is not resolved until the separated absorption charge and multiplication (SACM) structure APD is proposed. On the one hand, the UV light can be effectively absorbed by the absorption layer, and on the other, the high internal gain can be obtained in the high-field multiplication layer by way of the impact ionization process. The impact ionization process in multiplication layer can be terminated by the charge control layer [13, 14]. The advantage for the SACM structure arises from the reduced noise, because only a single type of photo-generated carriers with larger ionization rate can be injected into the multiplication layer [15, 16]. To obtain a high detectivity for weak ultraviolet signal, SACM APDs with large active detection area shall be fabricated [17]. However, the increased device size is accompanied by the significantly enhanced surface leakage current and bulk leakage current. Thus, this not only imposes a strict requirement on the quality of SiC epitaxial wafer, but also reflects a great challenge to the device fabrication process and device design. In the past few decades, Cree Company has greatly promoted epitaxial growth technology for SiC films, which thus has further led to the continuous improvement for crystalline quality. Most recently, Zhou et al. have proposed a variable-temperature photoresist reflow technique to create very smooth sidewalls for the beveled 4H-SiC APD mesa [18], which enables a high multiplication gain of over 10^6^ and a low dark current of ~ 0.2 nA/cm^2^. Nevertheless, the previous research has more focused on improving the material quality and optimizing the fabrication technology for SACM APDs [19–21], while the impact of structural design on photo-generated carrier transport and photocurrent detectivity has been rarely discussed till now. Hence, in this letter, we systematically investigate the optoelectronic performance for large-area 4H-SiC-based SACM ultraviolet APDs with different structural designs. Meanwhile, insightful physical images and discussions are also provided. We believe the findings in this work are useful for researchers to optimize 4H-SiC APDs a lower cost.

As it is known, the large-scale carrier multiplication is generated when the impact ionization takes place, which, nevertheless, is strongly influenced by the thickness for the multiplication layer and the doping concentration for the charge control layer. The very strong electric field is produced in the multiplication layer for enabling the impact ionization. The electric field can be terminated by the charge control layer because of the larger doping concentration therein. In addition, we also find that, by modulating the energy band between the absorption layer and the charge control layer, we are able to adjust the spectral responsivity. A reasonable adoption of a positive beveled mesa can achieve a significant reduction in the sidewall surface electric field, which is helpful to suppress the dark current and edge breakdown. Detailed analysis and discussions will be conducted subsequently.

## Research Methods and Physics Models

**Fig. 1:**
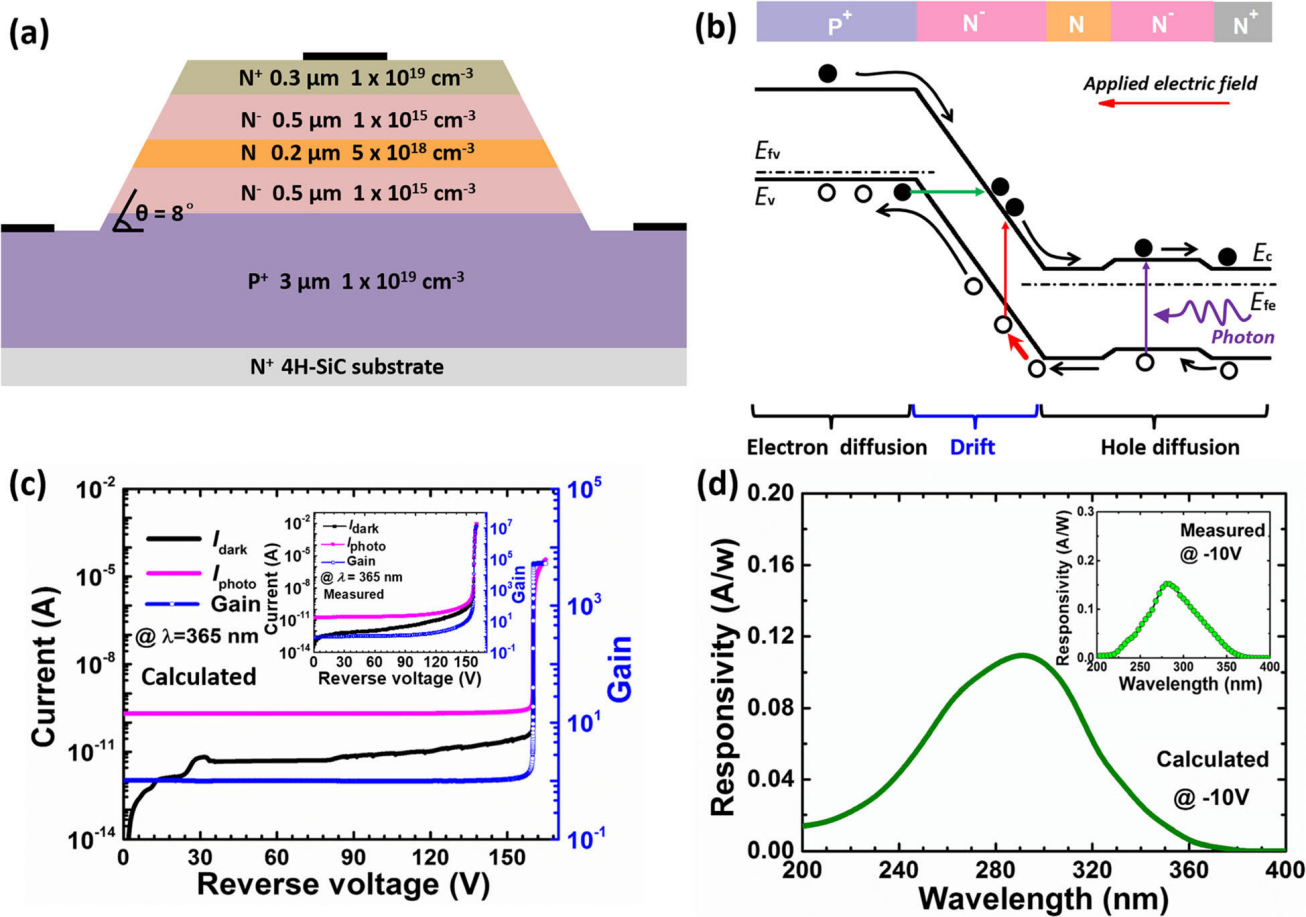
**a** Schematic cross-sectional structure (not drawn in scale), **b** schematic energy band diagram under reverse bias for standard 4H-SiC SACM APD, **c** calculated current-voltage characteristics and the multiplication gain, and **d** calculated spectral response characteristics at the reverse voltage of 10 V for standard 4H-SiC SACM APD. Inset figure in **c** shows the measured current-voltage characteristics and the multiplication gain. Inset figure in **d** shows measured spectral response characteristics for standard 4H-SiC SACM APD biased at − 10 V

Figure 1a shows the schematic cross-sectional view for a standard 4H-SiC SACM APD employed in this work, which possess n^+^-type 4H-SiC layer as the substrate. Then, the architectural stack comprises a 3-μm-thick p^+^-type layer (*N*_*a*_ = 1 × 10^19^ cm^−3^) serving as the p-type ohmic contact layer, a 0.5-μm-thick n^−^-type multiplication layer (*N*_*d*_ = 1 × 10^15^ cm^−3^) for carrier multiplication, a 0.2-μm-thick n-type charge control layer (*N*_*d*_ = 5 × 10^18^ cm^−3^) for terminating the impact ionization process, and a 0.5-μm-thick n^−^-type absorption layer (*N*_*d*_ = 1 × 10^15^ cm^−3^) to absorb the incoming photons. On top of the device structure, there is a 0.3-μm-thick n^+^-type ohmic contact layer (*N*_*d*_ = 1 × 10^19^ cm^−3^). A positive bevel angle (*θ* = 8°) is created for the mesa structure to suppress the edge breakdown [22, 23]. The diameter for the 4H-SiC SACM APDs is 800 μm. Both the cathode and anode contacts are considered as ideal ohmic contacts in our calculations. According to Fig. 1c and d, our results illustrate that the calculated results for the aforementioned standard 4H-SiC SACM APD structure exhibit the dark current of 2.5 nA/cm^2^, the breakdown voltage of 161.6 V, and the peak responsivity of 0.11 A/W at the wavelength of 280 nm. The calculated current–voltage characteristics and responsivity for standard 4H-SiC SACM APD are consistent with the experimental data. This fully proves that the impact ionization, 4H-SiC material absorption coefficient, Poisson’s equation, current continuity equation, and drift-diffusion equations used in this work are reasonable. Here, we take the structure in Fig. 1a as the research benchmark while the investigated variables include beveled mesa angle, the thickness, and the doping concentration for each layer.

To better understand the impact of different structural variables on the photoelectric properties for 4H-SiC SACM APDs, Fig. 1b shows the schematic energy band diagram under reverse bias. The photo-generated electron-hole pairs in the absorption layer will diffuse into the multiplication layer by way of the charge control layer. After the photo-generated electron-hole pairs reach the multiplication region, they will be separated by the depleted electric field. Holes will then experience multiplication process in the multiplication layer, which gives rise to internal current gain. Meanwhile, the photo-generated electrons will return to the cathode and are converted into current without experiencing impact ionization. Important factors that influence the photocurrent include the electric field profiles in the multiplication layer, the energy band alignment in the charge control layer, the absorption layer, and the n-type ohmic contact layer, in which any energy barrier can hinder the carrier transport. It is also worth noting that the electric field profiles in the multiplication layer can be determined by the doping concentrations for both the multiplication layer and the charge control layer. Meanwhile, the beveled mesa angle is also substantially associated with the electric field distribution. We shall also pay attention to the thickness for each layer to ensure the high-efficiency carrier diffusion process. Therefore, it is important to systematically study these key structural parameters for optimizing the device performance.

The numerical investigations are conducted by APSYS, which can solve current continuity equations, Poisson’s equations, and drift-diffusion equations consistently with proper boundary conditions. Both impact ionization and Zener tunneling processes have been included in drift-diffusion equations. The carrier-carrier scattering for the carrier transport process has also been included in low field mobility model. The Shockley-Read-Hall (SRH) recombination lifetime is assumed to 1 μs [24]. Specifically, the field (*E*) dependence of the impact ionization coefficients for electrons (*α*_*n*_) and holes (*β*_*p*_) for 4H-SiC-based layers can be expressed by Chynoweth formulas (1) and (2) as follows, respectively [25]:

1 $$ {\alpha}_n=1.98\times {10}^6\exp \left[-{\left(\frac{9.46\times {10}^6}{E}\right)}^{1.42}\right]{\mathrm{cm}}^{-1} $$

2 $$ {\beta}_p=4.38\times {10}^6\exp \left[-{\left(\frac{1.14\times {10}^7}{E}\right)}^{1.06}\right]{\mathrm{cm}}^{-1} $$

The absorption coefficient (∂) in terms of different wavelengths (λ) for 4H-SiC material is calculated by the following Eq. (3) [26]:

3 $$ \partial =-790.3+18.2\uplambda -0.17{\uplambda}^2+8.57\times {10}^{-4}{\uplambda}^3-2.39\times {10}^{-6}{\uplambda}^4+3.53\times {10}^{-9}{\uplambda}^5-2.16\times {10}^{-12}{\uplambda}^6 $$

The other material parameters used in the numerical models can be found elsewhere [27]. The calculations are based on finite element method, which requires users to properly adjust the mesh distributions for making accurate calculations.

## Results and Discussions

### Impact of the Structural Parameters for the n-Type Ohmic Contact Layer on the Photoelectric Performance

In order to probe the effect of the thickness and doping concentration for the n-type ohmic contact layer on the photoelectric performance, we design reference device, devices L1 to L4, and devices A1 to A4, respectively. Note that reference device is the basic SACM APD structure as it is shown in Fig. 1a. Other proposed APDs are identical to the reference device except the n-type SiC ohmic contact layer, the detailed structural information for which is presented in Table 1.

**Table 1:** Structural parameters for the n-type contact layer of the investigated devices

Structural information for the n-type contact layer			
Device number	Doping type, doping concentration, thickness	Device number	Doping type, doping concentration, thickness
Device L1	N^+^, 1 × 10^19^ cm^−3^, 0.1 μm	Device A1	N^+^, 1 × 10^18^ cm^−3^, 0.3 μm
Device L2	N^+^, 1 × 10^19^ cm^−3^, 0.2 μm	Device A2	N^+^, 5 × 10^18^ cm^−3^, 0.3 μm
Reference device	N^+^, 1 × 10^19^ cm^−3^, 0.3 μm	Reference device	N^+^, 1 × 10^19^ cm^−3^, 0.3 μm
Device L3	N^+^, 1 × 10^19^ cm^−3^, 0.4 μm	Device A3	N^+^, 2 × 10^19^ cm^−3^, 0.3 μm
Device L4	N^+^, 1 × 10^19^ cm^−3^, 0.5 μm	Device A4	N^+^, 5 × 10^19^ cm^−3^, 0.3 μm

**Fig. 2:**
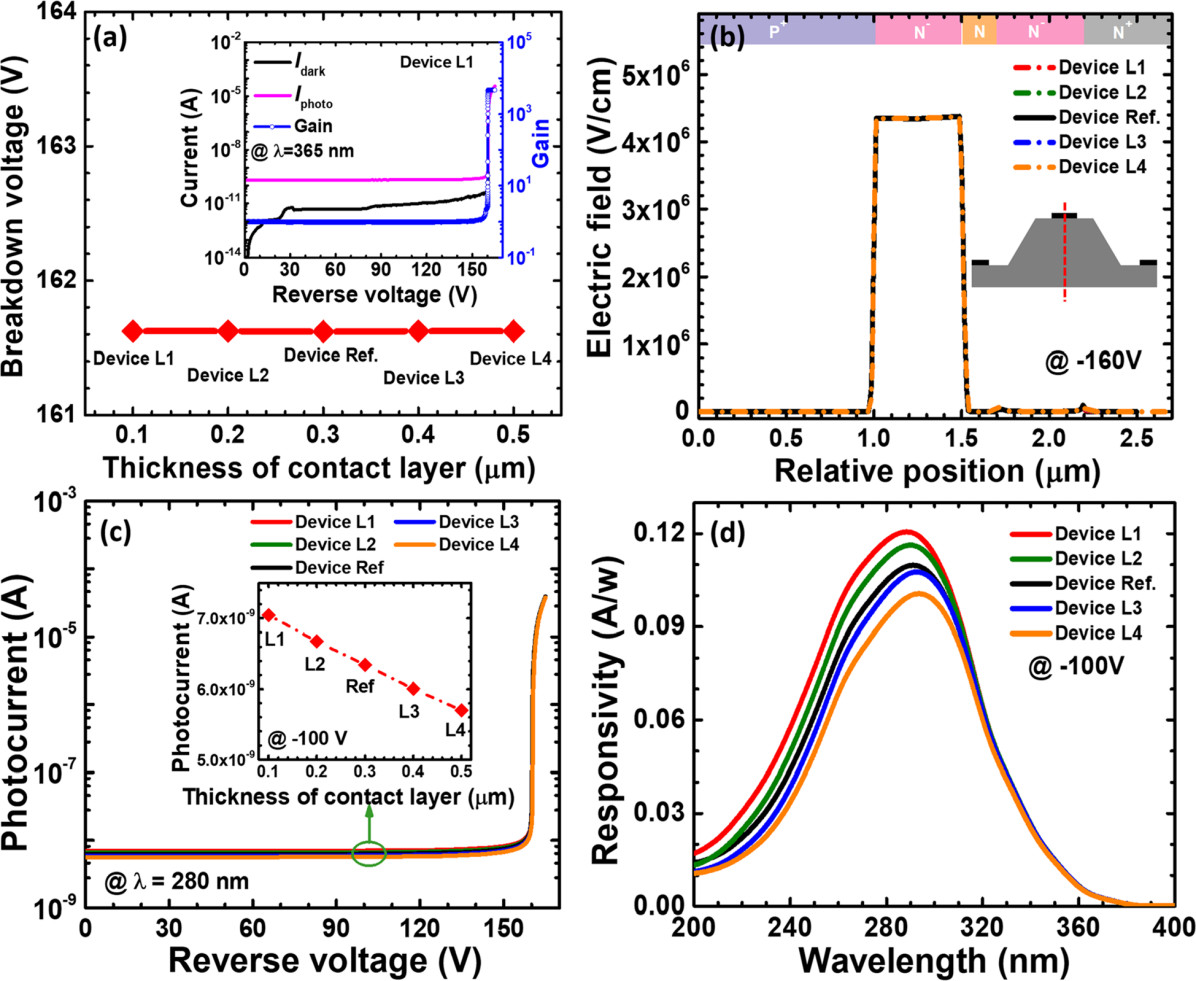
**a** Breakdown voltage, **b** vertical electric field distribution of the SACM APDs biased at − 160 V, **c** photocurrent-voltage characteristics under 280 nm illumination, and **d** spectral response characteristics of the SACM APDs biased at − 100 V for reference device and devices L1 to L4 with different thicknesses of n-type ohmic contact layer, respectively. Inset figure in **a** shows the calculated current-voltage characteristics and the multiplication gain for device L1. Inset figure in **c** shows photocurrent for reference device and devices L1 to L4 biased at − 100 V

We firstly show the breakdown voltage in terms of the thickness for the n-type SiC ohmic contact layer in Fig. 2a, i.e., devices L1 to L4. The inset for Fig. 2a selectively demonstrates the dark current, the photocurrent under the illumination of 365 nm, and the gain for device L1. For device L1, the breakdown voltage is ~ 161.6 V and the dark current density remains at the level of ~ 2.5 nA/cm^2^ when bias is lower than 161.6 V. Note that the breakdown voltage is obtained at the current of 10^−5^ A. The dark current increases when the impact ionization process occurs. The photocurrent level becomes high in the linear region when the 365nm illumination shines onto the device, and this shows the multiplication gain can be over 10^3^ for device L1 at the reverse voltage of 161.6 V. The avalanche breakdown voltage for the five investigated APDs is summarized in Fig. 2a. From Fig. 2a, we can get that the thickness for the n-type ohmic contact layer has negligible effect on the breakdown voltage. To reveal the underlying mechanism for the observations, we calculate and show the vertical electric field distribution for reference device and devices L1 to L4 in Fig. 2b, which illustrates that the charge control layer confines the boundary for the depletion region and the electric field in the multiplication layer. Therefore, we can speculate that the n-type ohmic contact layer will not affect the electric field profiles in the multiplication layer and this is proven in Fig. 2b. The observations in Fig. 2b well interprets the identical breakdown voltage in Fig. 2a for reference device and devices L1 to L4. Next, we show the photo-generated current for the five devices in Fig. 2c. For better resolution, we collect the photo-generated current at the bias of 100 V which are shown in the inset for Fig 2c. We can see that the photo-generated current decreases with the increasing thickness for the n-type ohmic contact layer. A too thick n-type ohmic contact layer will cause the photo-generated carriers to have nonradiative recombination and correspondingly reduces the diffusion current. With the photo-generated current, we can get the spectral responsivity for reference device and devices L1 to L4 at the reverse voltage of 100 V in Fig. 2d. The peak response wavelength for the five investigated devices is centered at 280 nm. The responsivity decreases with increasing thickness of the n-type contact layer, which agrees with the inset for Fig. 2c. Therefore, we summarize here that the thickness for the n-type 4H-SiC ohmic contact layer shall be properly thin to avoid the increased nonradiative recombination and the reduced diffusion current.

**Fig. 3:**
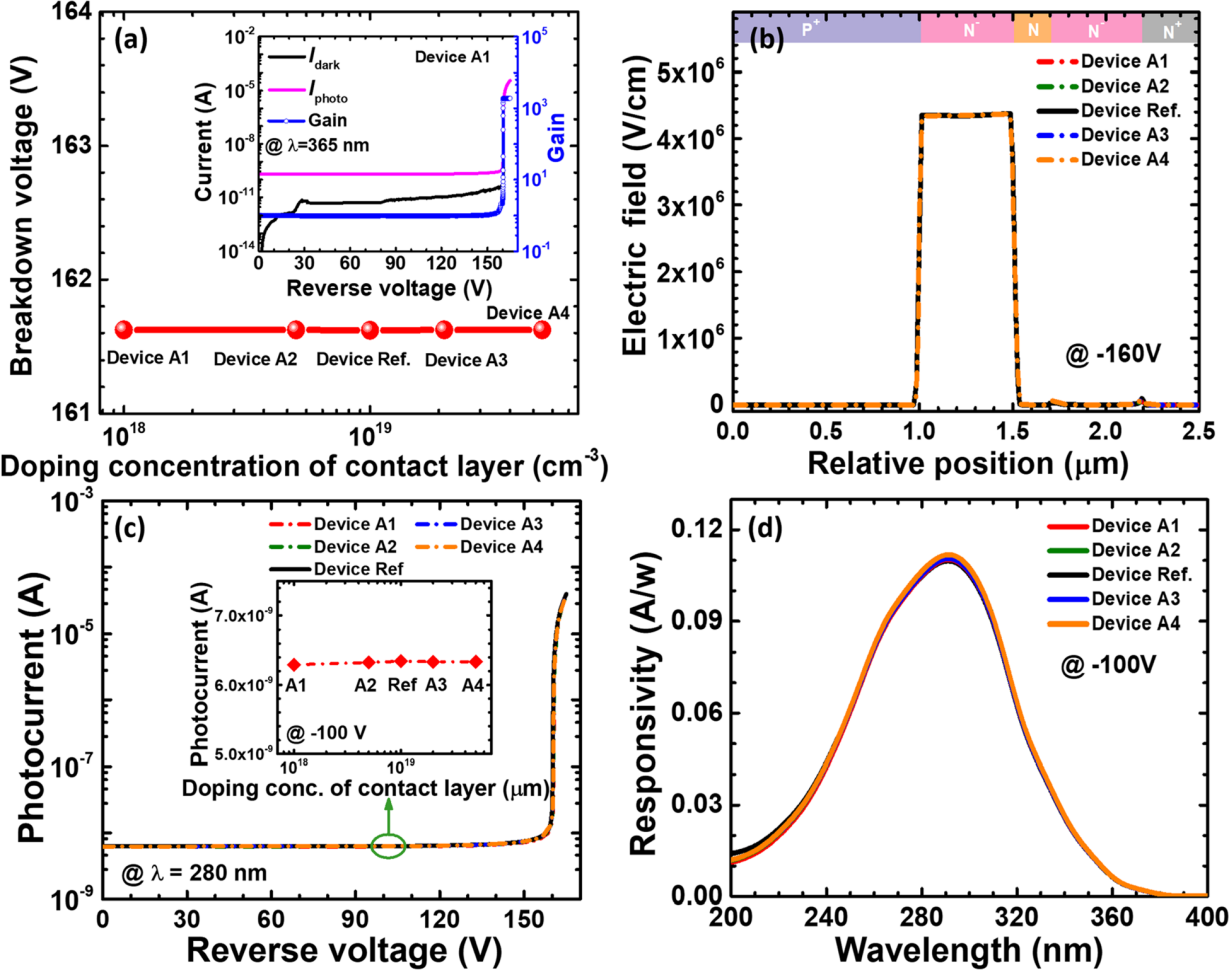
**a** Breakdown voltage, **b** vertical electric field distribution of the SACM APDs biased at − 160 V, **c** photocurrent-voltage characteristics under 280 nm illumination, and **d** spectral response characteristics of the SACM APDs biased at − 100 V for reference device and devices A1 to A4 with different doping concentration of n-type ohmic contact layer, respectively. Inset figure in **a** shows the calculated current-voltage characteristics and the multiplication gain for device A1. Inset figure in **c** shows photocurrent for reference device and devices A1 to A4 biased at − 100 V

Next, we investigate the breakdown voltage as a function of the doping concentration in the n-type ohmic contact layer by analyzing reference device and devices A1 to A4 in Fig. 3a. The inset in Fig. 3a presents the dark current, the photo-generated current, and the gain in terms of the applied bias for device A1. The breakdown voltage is defined when the current reaches 10^−5^ A. According to Fig. 3a, the breakdown voltage hardly depends on the doping concentration in the n-type 4H-SiC ohmic contact layer. As has been demonstrated earlier, the charge control layer can effective confine the depletion region and the electric field in the multiplication layer. Hence, the variation for the doping concentration in the n-type ohmic contact layer does not affect the electric field distribution inside the device [see Fig. 3b]. We then calculate and show photo-generated current in terms of the applied bias for reference device and devices A1 to A4 in Fig. 3c. It can be seen from Fig. 3c that the doping concentration of the n-type ohmic contact layer has negligible effect on the photocurrent biased. The spectral responsivity at different wavelengths for the five investigated devices is shown in Fig. 3d. The data are computed at the reverse voltage of 100 V. The wavelength with maximum responsivity of ~ 0.11 A/W is 280 nm. Being consistent with Fig. 3c, the responsivity is less dependent on the doping concentration in the n-type ohmic contact layer. Therefore, we conclude that the responsivity is more influenced by thickness than the doping concentration for the n-type ohmic contact layer for 4H-SiC SACM APDs. We also suggest increasing the carrier diffusion length for the purpose of enhancing the responsivity.

### Impact of the Structural Parameters for the Absorption Layer on the Photoelectric Performance

In this section, the impact of the thickness and doping concentration for the absorption layer on the photoelectric performance for 4H-SiC-based SACM APDs is studied. The detailed structural information of the absorption layer for SACM APDs is summarized and shown in Table 2. Devices M1 to M4 and devices B1 to B4 are structurally identical to reference device except the absorption layer. Devices M1 to M4 have different thicknesses while devices B1 to B4 possess various doping concentrations for the absorption layer.

**Table 2:** Structural parameters for the absorption layer of the investigated devices

Structural information for the absorption layer			
Device number	Doping type, doping concentration, thickness	Device number	Doping type, doping concentration, thickness
Device M1	N^−^, 1 × 10^15^ cm^−3^, 0.2 μm	Device B1	N^−^, 5 × 10^14^ cm^−3^, 0.5 μm
Device M2	N^−^, 1 × 10^15^ cm^−3^, 0.4 μm	Reference device	N^−^, 1 × 10^15^ cm^−3^, 0.5 μm
Reference device	N^−^, 1 × 10^15^ cm^−3^, 0.5 μm	Device B2	N^−^, 5 × 10^15^ cm^−3^, 0.5 μm
Device M3	N^−^, 1 × 10^15^ cm^−3^, 0.6 μm	Device B3	N^−^, 1 × 10^16^ cm^−3^, 0.5 μm
Device M4	N^−^, 1 × 10^15^ cm^−3^, 0.8 μm	Device B4	N^−^, 5 × 10^16^ cm^−3^, 0.5 μm

**Fig. 4:**
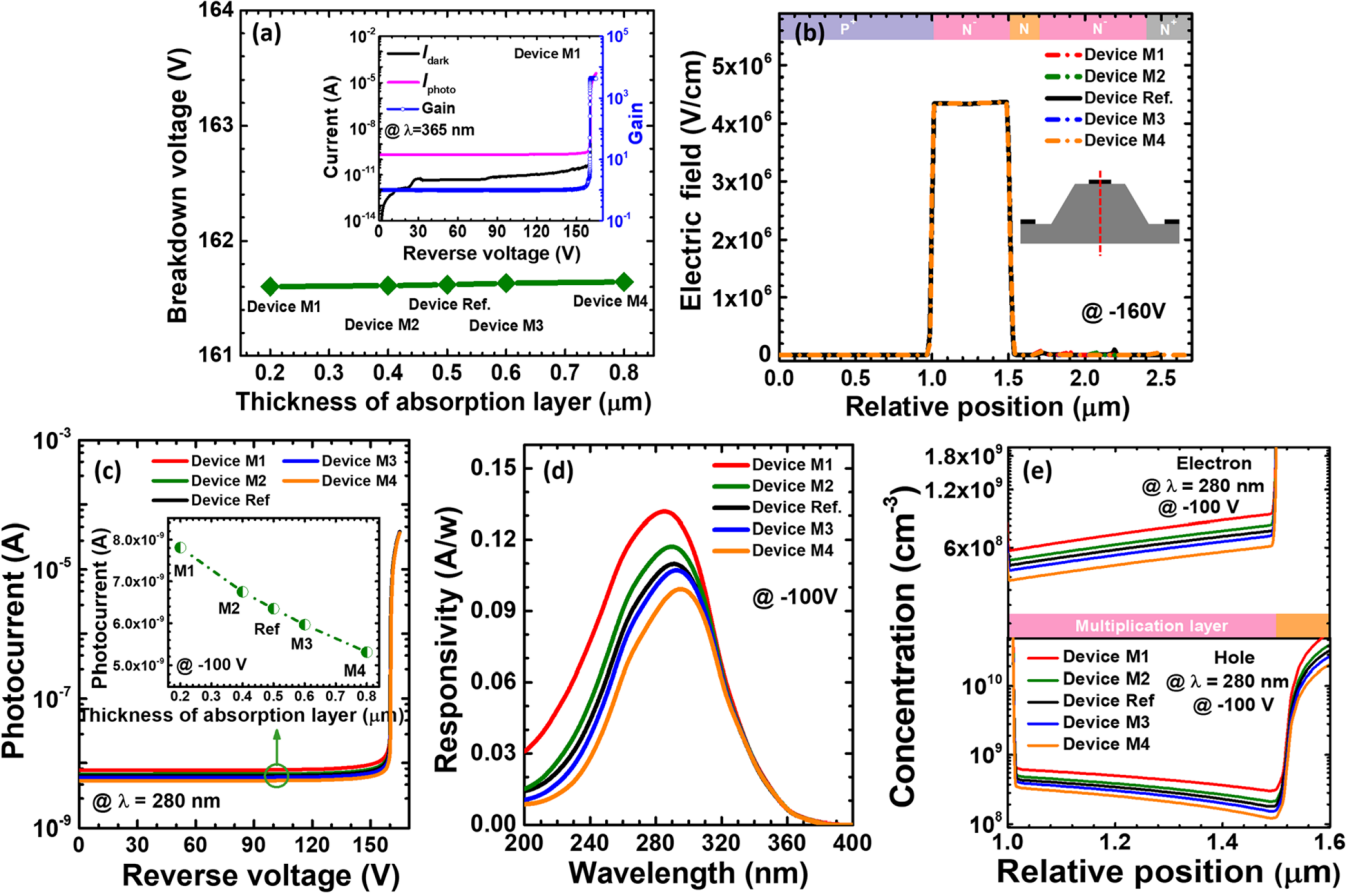
**a** Breakdown voltage, **b** vertical electric field distribution of the SACM APDs biased at − 160 V, **c** photocurrent-voltage characteristics under 280 nm illumination, **d** spectral response characteristics, and **e** carrier concentration profiles in the multiplication layer of the SACM APDs biased at − 100 V for reference device and devices M1 to M4 with different thicknesses of absorption layer, respectively. Inset figure in **a** shows the calculated current-voltage characteristics and the multiplication gain for device M1. Inset figure in **c** shows photocurrent for reference device and devices M1 to M4 biased at − 100 V

By using reference device and devices M1 to M4, Fig. 4a shows the breakdown voltage in terms of different thicknesses for the absorption layer. For the purpose of demonstration, we calculate and present the dark current, the photo-generated current, and the gain as a function of the applied bias for device M1 in the inset of Fig. 4a. The breakdown voltage is collected when the current is 10^−5^ A. We can see that the breakdown voltage hardly depends on the thickness for the absorption layer. It is known that the breakdown voltage is strongly subject to the electric field intensity in the lightly doped multiplication layer, and hence, Fig. 4b shows the vertical electric field distribution for the five studied devices at the reverse bias of − 160 V. The electric field distributions for reference device and devices M1 to M4 are exactly the same, which confirms the conclusion in Fig. 4a. We subsequently demonstrate the photo-generated current and the responsivity in Figs. 4c and d, respectively. Both the photo-generated current [see the inset for Fig. 4c] and the responsivity show the trend of decrease with the increased thickness for the absorption layer. To further address the underlying mechanism, we also calculate and show the carrier distribution within the multiplication layer in Fig. 4e when the reverse bias is 100 V for the five investigated devices. We can see that both the electron and hole concentration levels decrease with the increasing absorption layer thickness, which is attributed to the enhanced nonradiative recombination when the absorption layer becomes thick. The nonradiative recombination consumes carriers, thus suppressing the diffusion current and the responsivity. Here, in order to avoid the carrier consumption by nonradiative recombination, we suggest that the absorption layer cannot be too thick for obtaining 4H-SiC SACM APDs with high detectivity.

**Fig. 5:**
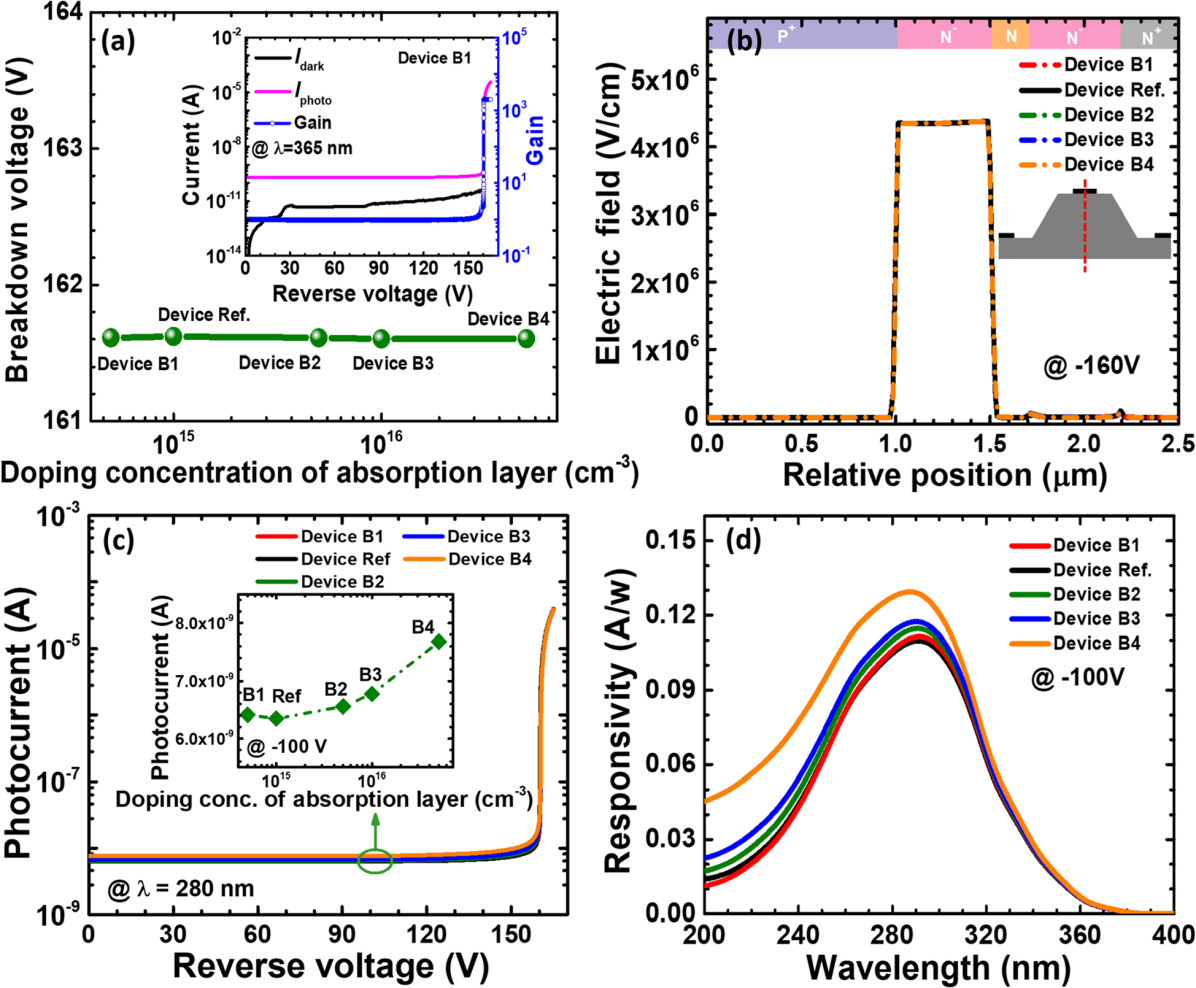
**a** Breakdown voltage, **b** vertical electric field distribution of the SACM APDs biased at − 160 V, **c** photocurrent-voltage characteristics under 280 nm illumination, and **d** spectral response characteristics of the SACM APDs biased at − 100 V for reference device and devices B1 to B4 with different doping concentration of absorption layer, respectively. Inset figure in **a** shows the calculated current-voltage characteristics and the multiplication gain for device B1. Inset figure in **c** shows photocurrent for reference device and devices B1 to B4 biased at − 100 V

Besides the absorption layer thickness, the doping concentration for the absorption layer also has a significant impact on the device performance. We then calculate and show the breakdown voltage for reference device and devices B1 to B4 in Fig. 5a. The breakdown voltage is defined when the current is 10^−5^ A as shown in the inset of Fig 5a. It can be seen from Fig. 5a that the doping concentration for the absorption layer has no significant effect on the breakdown voltage. It can be further proven by the vertical one-dimensional electric field distributions in Fig. 5b, such that the doping concentration for the absorption layer does not significantly change the electric field profiles in the multiplication layer. We also present the photo-generated current at the wavelength of 280 nm for the studied devices in Fig. 5c, which indicates the improved photo-generated current when the doping concentration in the absorption layer increases. Agreeing well with Fig. 5c, the wavelength-dependent responsivity in Fig. 5d is also favored as the doping concentration for the absorption layer increases, e.g., device B4.

**Fig. 6:**
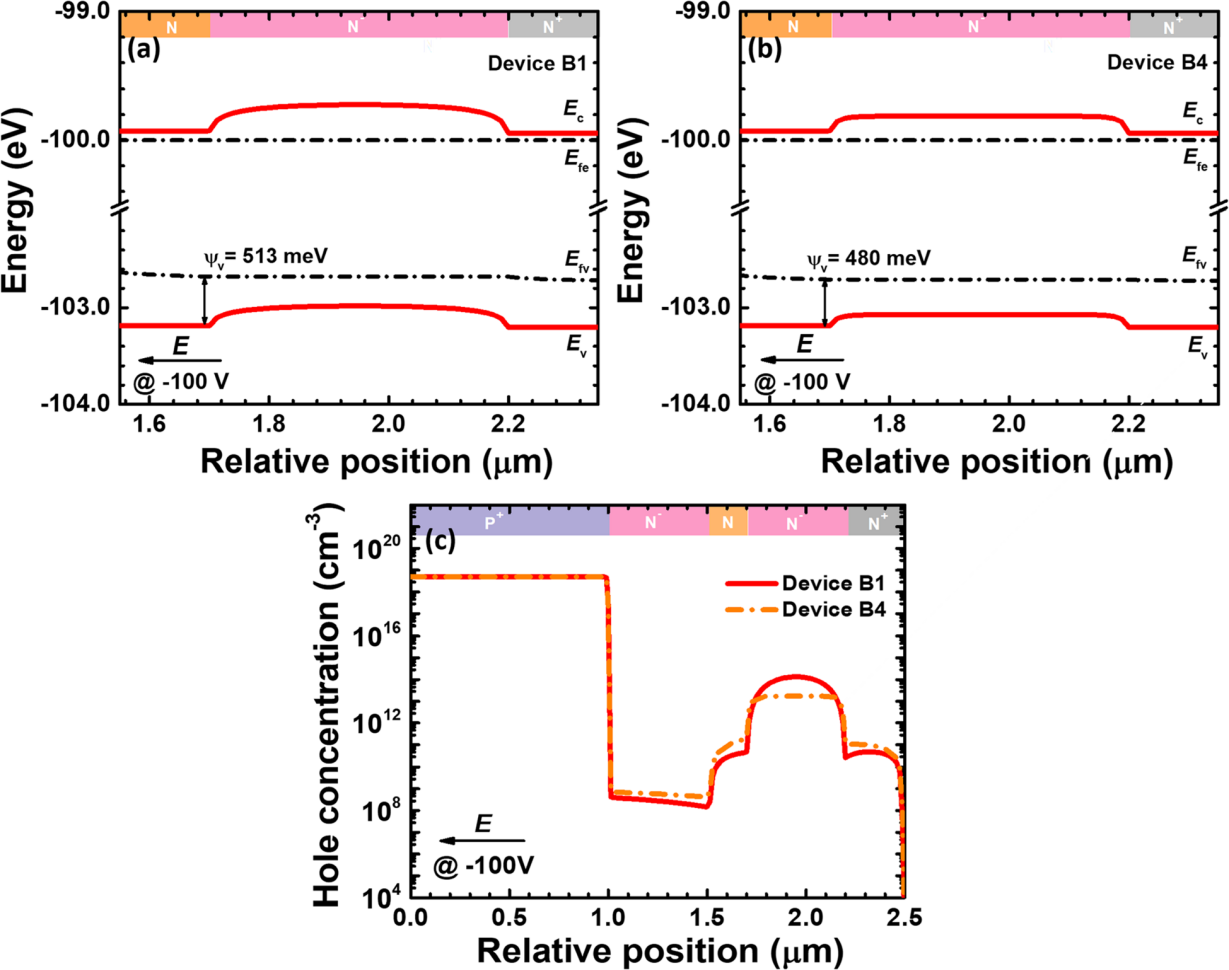
Energy band diagrams of charge control layer, absorption layer and n-type ohmic contact layer for **a** device B1 and **b** device B4, **c** hole concentration profiles under 280 nm illumination for device B1 and B4. Data are calculated at the reverse voltage of 100 V

In order to show the in-depth origin for the enhanced responsivity for device B4, we show the energy band profiles for the charge control layer, the absorption layer, and the n-type ohmic contact layer for devices B1 and B4 in Figs. 6a and b, respectively. Here, it is worth mentioning that the doping concentrations for the charge control layer and the n-type ohmic contact layer are 5 × 10^18^ cm^−3^ and 1 × 10^19^ cm^−3^, respectively. Therefore, the lower doping concentration for the absorption layer can generate a built-in electric field and create the energy barriers at interfaces of charge control layer/absorption layer/n-type ohmic contact layer [28]. The energy barriers can retard the diffusion for the photo-generated carriers into the multiplication layer. A very convenient method that can reduce the barriers is to increase the doping concentration in the absorption layer. As a result, the effective valence band barrier values *ψ*_*v*_ for the charge control layer are 513 meV and 480 meV for devices B1 and B4, respectively. It is witnessed that the increased doping concentration for the absorption layer promotes the transport for the photo-generated holes [see Fig. 6c]. The impact ionization will become strong once more photo-generated holes can be injected into the multiplication region, which correspondingly results in the increased photo-generated current and the responsivity.

### Impact of the Structural Parameters for the Charge Control Layer on the Photoelectric Performance

To probe the impact of the thickness and doping concentration of the charge control layer on photoelectric performance, we set different architectural information for the charge control layer as shown in Table 3. Devices N1 to N4 and devices C1 to C4 are different from reference device only in the charge control layer. Different doping concentrations and layer thicknesses are adopted for devices N1 to N4 and C1 to C4.

**Table 3:** Structural parameters for the charge control layer of the investigated devices

Structural information for the charge control layer			
Device number	Doping type, doping concentration, thickness	Device number	Doping type, doping concentration, thickness
Device N1	N, 5 × 10^18^ cm^−3^, 0.1 μm	Device C1	N, 2 × 10^17^ cm^−3^, 0.2 μm
Reference device	N, 5 × 10^18^ cm^−3^, 0.2 μm	Device C2	N, 5 × 10^17^ cm^−3^, 0.2 μm
Device N2	N, 5 × 10^18^ cm^−3^, 0.3 μm	Device C3	N, 2 × 10^18^ cm^−3^, 0.2 μm
Device N3	N, 5 × 10^18^ cm^−3^, 0.5 μm	Reference device	N, 5 × 10^18^ cm^−3^, 0.2 μm
Device N4	N, 5 × 10^18^ cm^−3^, 0.7 μm	Device C4	N, 7 × 10^18^ cm^−3^, 0.2 μm

**Fig. 7:**
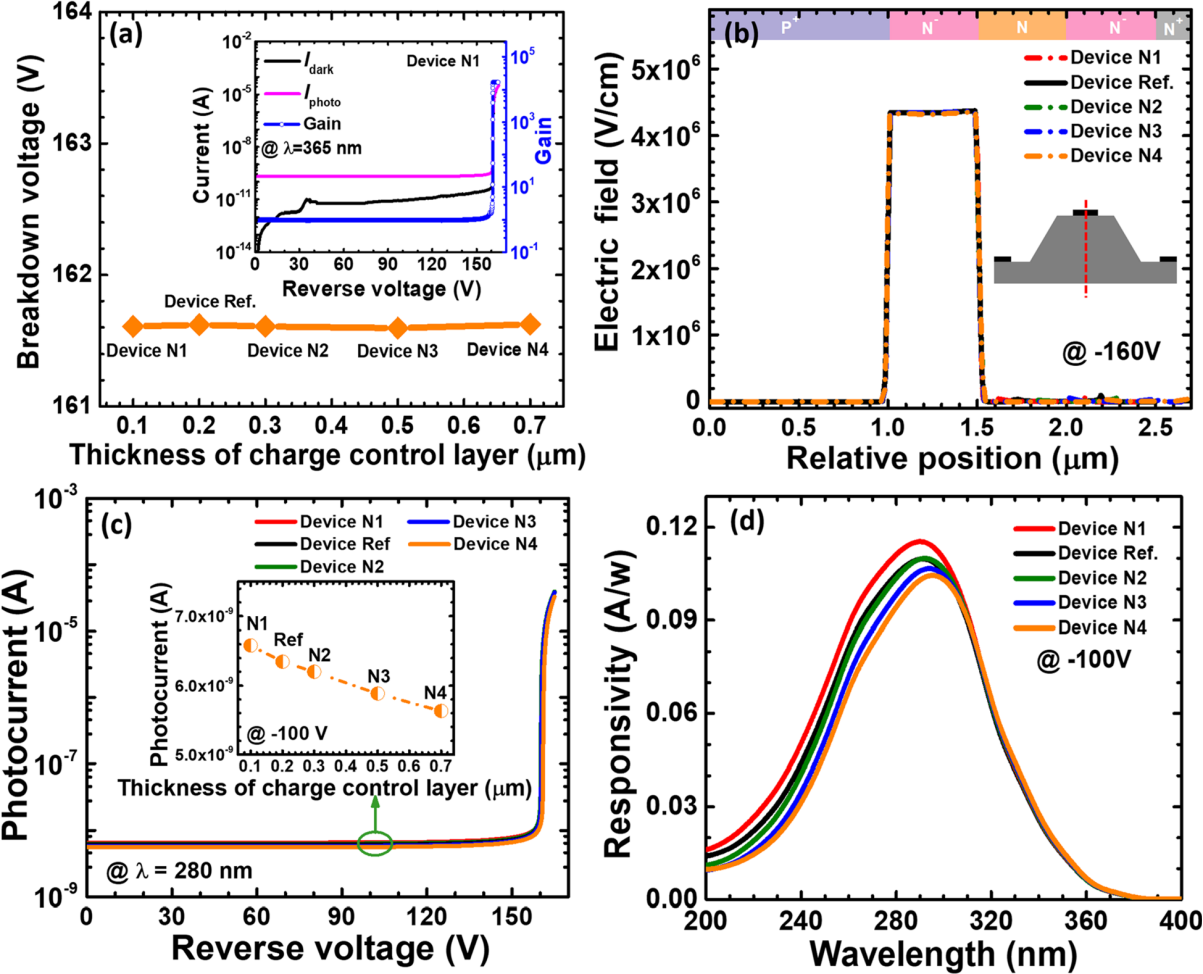
**a** Breakdown voltage, **b** vertical electric field distribution of the SACM APDs biased at − 160 V, **c** photocurrent-voltage characteristics under 280 nm illumination, and **d** spectral response characteristics of the SACM APDs biased at − 100 V for reference device and devices N1 to N4 with different thicknesses of charge control layer, respectively. Inset figure in **a** shows the calculated current-voltage characteristics and the multiplication gain for device N1. Inset figure in **c** shows photocurrent for reference device and devices N1 to N4 biased at − 100 V

As has been mentioned previously, the electric field that enables the impact ionization and the avalanche breakdown is mainly confined in the multiplication layer. The breakdown voltage as a function of the thickness for the charge control layer in Fig. 7a infers that the thickness of the charge control layer has very slight effect on the carrier multiplication process. This is further proven by showing Fig. 7b. Figure 7c demonstrates the photo-generated current in terms of the applied bias for reference device and devices N1 to N4. The photo-generated current becomes low once the charge control layer thickness increased, which also translates the smaller responsivity with the increasing the thickness for the charge control layer. We also attribute to the enhanced nonradiative recombination that consumes carriers and suppresses the diffusion current.

**Fig. 8:**
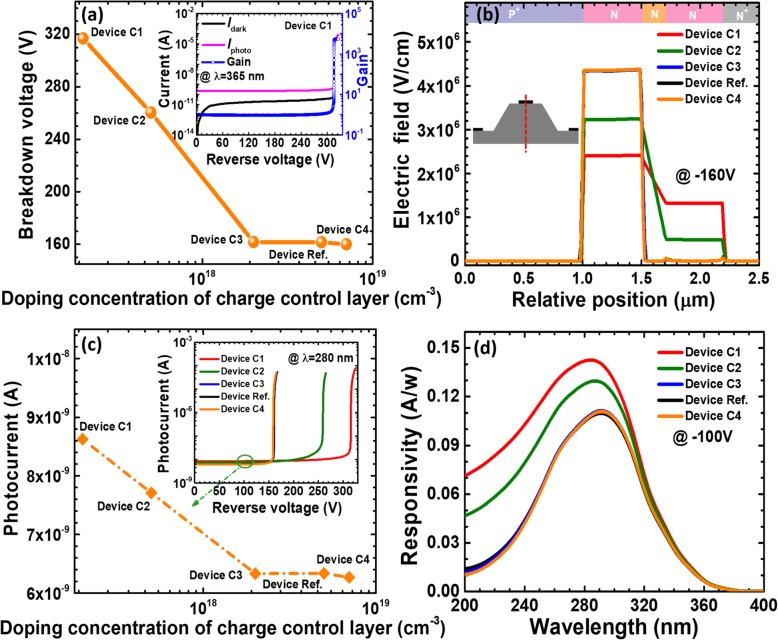
**a** Breakdown voltage, **b** vertical electric field distribution of the SACM APDs biased at − 160 V, **c** photocurrent biased at − 100 V, and **d** spectral response characteristics of the SACM APDs biased at − 100 V for reference device and devices C1 to C4 with different doping concentration of charge control layer, respectively. Inset figure in **a** shows the calculated current-voltage characteristics and the multiplication gain for device C1. Inset figure in **c** shows photocurrent-voltage characteristics for reference device and devices C1 to C4

The role of the charge control layer is to confine the strong electric field and the carrier multiplication process within the multiplication layer. However, the depletion region width may be further extended as long as the doping concentration in the charge control layer decreases. The electric field profiles can then substantially affect the breakdown voltage, the photo-generated current, the gain, and the responsivity. Therefore, we design devices C1 to C4 in Table 3. According to Fig. 8a, as the doping concentration decreases, the breakdown voltage initially remains the same, and then the breakdown voltage increases when the doping concentration for the charge control layer is below 2 × 10^18^ cm^−3^. The inset of Fig. 8a indicates that the breakdown voltage is ~ 315 V for device C1 while the dark current also rises to 3.5 × 10^−11^ A compared with that for device N1. To reveal the origin for the observations in Fig. 8a, we calculate the vertical electric field distribution in Fig. 8b, which demonstrates that the electric field is mainly concentrated in the multiplication layer for reference devices and devices C3 and C4. However, the electric field and the depletion region penetrate into the charge control layer when the doping concentration for the charge control layer is lower than 2 × 10^18^ cm^−3^. The expansion of the depletion region for devices C1 and C2 helps to reduce the electric field intensity and thus the breakdown voltage is correspondingly increased for devices C1 and C2. The increased depletion region width will yield more space charge generation current, which thus results in an increased dark current, i.e., 3.5 × 10^− 11^ A and 5 × 10^− 11^ A for devices C1 and C2, respectively. We then show the photo-generated current at the wavelength of 280 nm in Fig. 8c. The 100V-biased spectral responsivity curves at different wavelengths for the five investigated devices are illustrated in Fig. 8d. Excellent agreement is obtained between Figs. 8c and d, such that the increased photo-generated current gives rise to the enhanced responsivity, i.e., devices C1 and C2. Other devices show similar photo-current level and the responsivity.

**Fig. 9:**
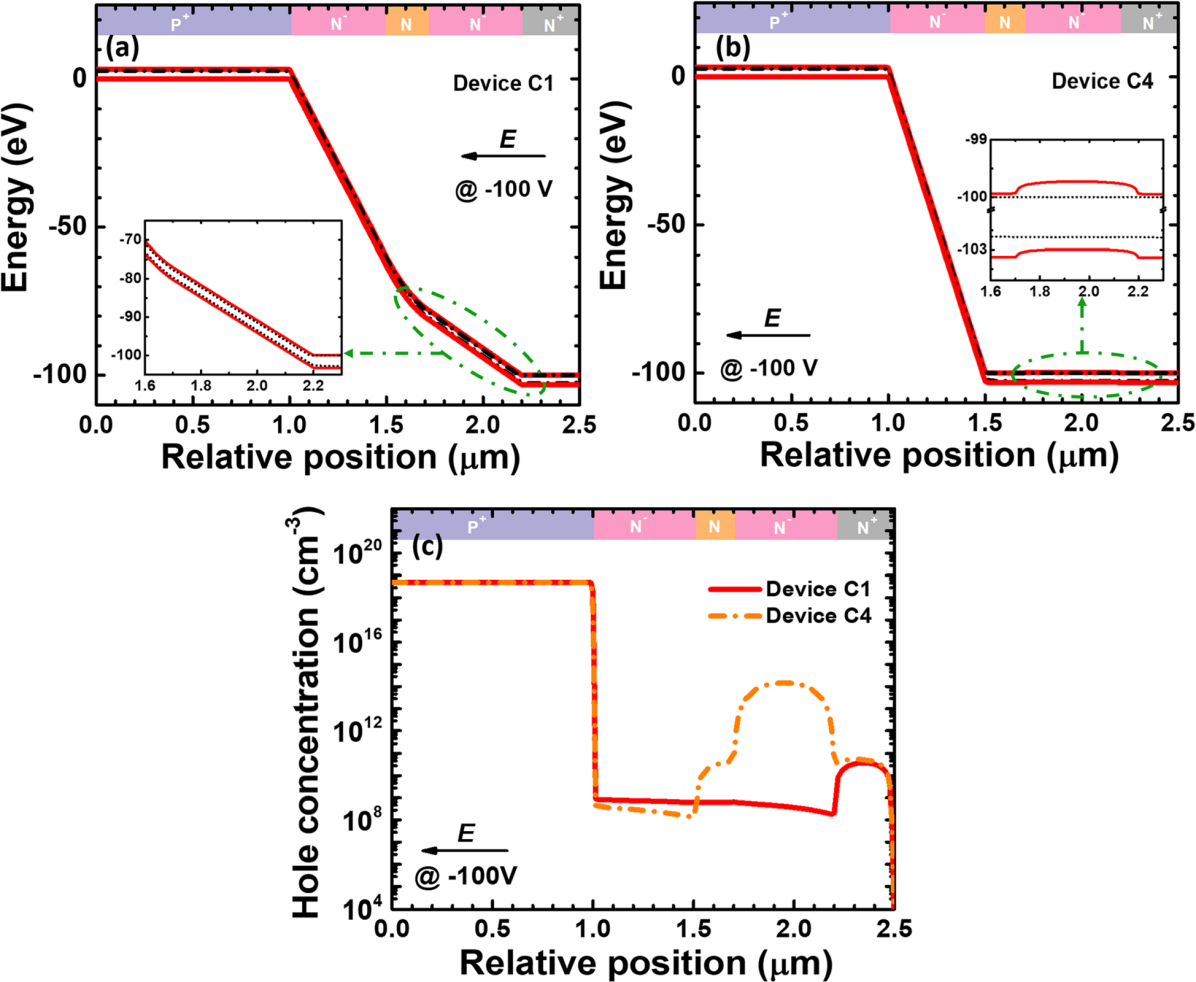
Energy band diagrams for **a** device C1 and **b** device C4, **c** hole concentration profiles for device C1 and C4. Data are calculated at the reverse voltage of 100 V. Insets for **a** and **b** show the local energy band diagrams for multiplication layer/charge control layer for devices C1 and C4, respectively

As has been interpreted previously, the energy band barrier height at the interface of multiplication layer/charge control layer can soundly affect the carrier diffusion. Due to the expansion of the depletion region for devices C1 and C2, the electric field in the depletion region will annihilate the energy barrier at the interface of multiplication layer/charge control layer [e.g., the inset for device C1 in Fig. 9a]. Meanwhile, we observe the valence band barrier at the interface of multiplication layer/charge control layer for device C4 according to the inset for Fig. 9b. The energy barrier will correspondingly retard the hole diffusion into the multiplication layer from the charge control layer. We also selectively compute and show the hole concentration profiles for device C1 and C4 in Fig. 9c. Because the interface of multiplication layer/charge control layer for device C1 no longer hinders the injection of photo-generated holes into the multiplication layer, more holes are limited in the charge control layer and the absorption layer for device C4. As a result, the hole concentration in the multiplication layer for device C1 is higher than that for device C4. Thus, the enhanced photo-generated current and the responsivity for device C1 are obtained when compared with device C4.

### Impact of the Structural Parameters for the Multiplication Layer on the Photoelectric Performance

The impact ionization and the carrier multiplication process take place in the multiplication layer, making the design for the multiplication layer essentially vital for 4H-SiC SACM APDs. Therefore, we look into the impact of the thickness and doping concentration for the multiplication layer on the photoelectric performance for SACM APDs. The detailed structural information of the multiplication layer for different SACM APDs are summarized and presented in Table 4. The only difference for the devices in Table 4 lies on the multiplication layer.

**Table 4:** Structural parameters for the multiplication layer for the investigated devices

Structural information for the multiplication layer			
Device number	Doping type, doping concentration, thickness	Device number	Doping type, doping concentration, thickness
Device P1	N^−^, 1 × 10^15^ cm^−3^, 0.3 μm	Device D1	N^−^, 5 × 10^14^ cm^−3^, 0.5 μm
Device P2	N^−^, 1 × 10^15^ cm^−3^, 0.4 μm	Reference device	N^−^, 1 × 10^15^ cm^−3^, 0.5 μm
Reference device	N^−^, 1 × 10^15^ cm^−3^, 0.5 μm	Device D2	N^−^, 5 × 10^15^ cm^−3^, 0.5 μm
Device P3	N^−^, 1 × 10^15^ cm^−3^, 0.6 μm	Device D3	N^−^, 1 × 10^16^ cm^−3^, 0.5 μm
Device P4	N^−^, 1 × 10^15^ cm^−3^, 0.7 μm	Device D4	N^−^, 5 × 10^16^ cm^−3^, 0.5 μm

**Fig. 10:**
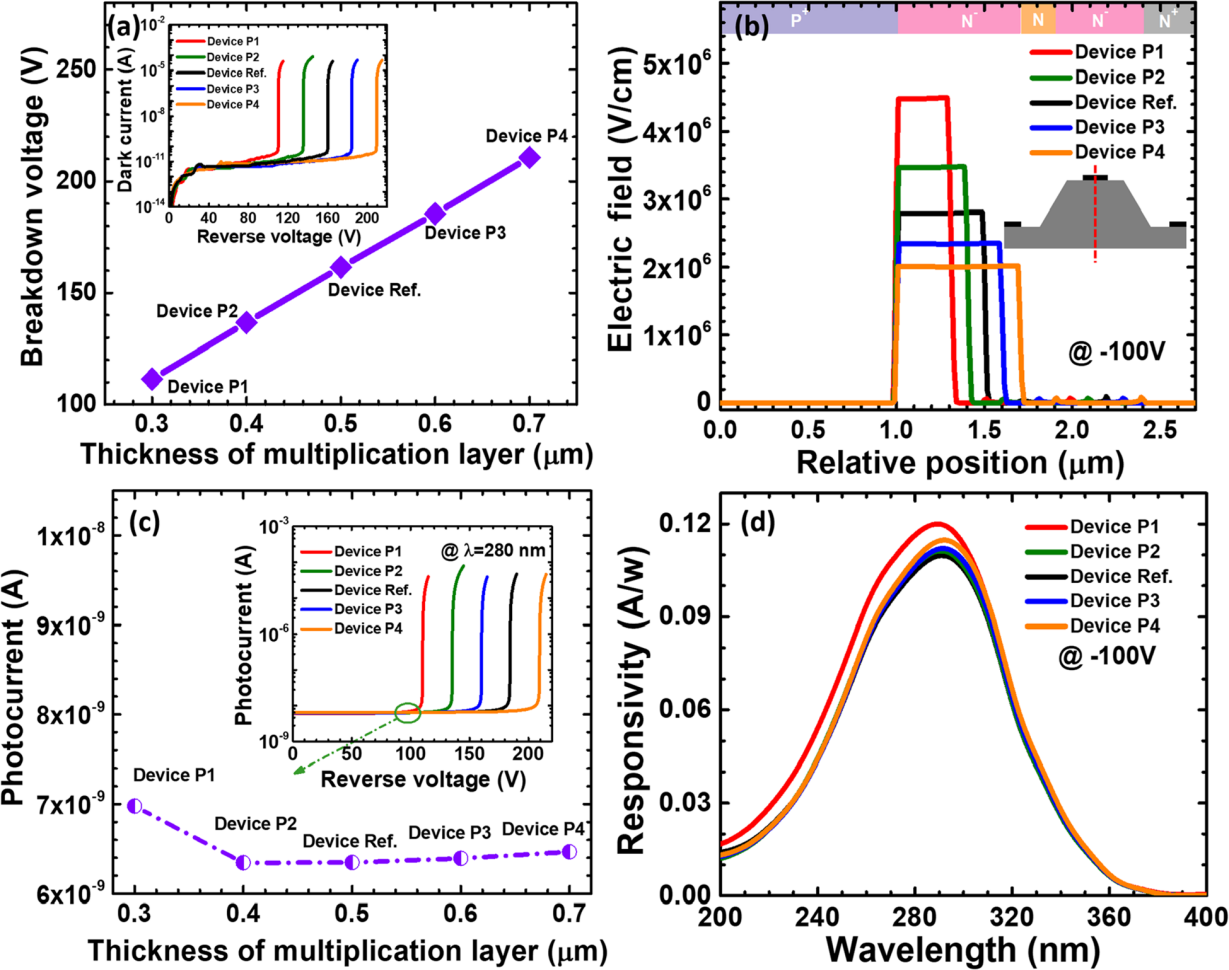
**a** Breakdown voltage, **b** vertical electric field distribution of the SACM APDs biased at − 160 V, **c** photocurrent biased at − 100 V, and **d** spectral response characteristics of the SACM APDs biased at − 100 V for reference device and devices P1 to P4 with different thicknesses of multiplication layer, respectively. Inset figure in **a** shows the calculated current-voltage characteristics for reference device and devices P1 to P4. Inset figure in **c** shows photocurrent-voltage characteristics under 280 nm illumination for reference device and devices P1 to P4

As Fig. 10a presents, the breakdown voltage is enhanced from 110 to 210 V when the multiplication layer thickness is increased from 0.3 to 0.7 μm. For the purpose of demonstration, the inset of Fig. 10a demonstrates the current in terms of the voltage for reference device and devices P1 to P4. This indicates that a thick multiplication layer helps to reduce the electric field intensity [see Fig. 10b] and increase the breakdown voltage. We then show the photo-generated current for the five devices in Fig. 10c. The photo-generated current increases slightly with increasing the thickness of the multiplication layer for devices P2 to P4, except that device P1 has the highest photocurrent. The spectral responsivity characteristics for the five investigated devices at the reverse voltage of 100 V are provided in Fig. 10d. The peak responsivity for reference device and devices P2 to P4 improves slightly as the thickness of the multiplication layer increases, and this is because the number of carriers generated by impact ionization increases when the depletion region width increases. Note that device P1 with the thinnest multiplication layer owns the highest peak responsivity at the wavelength of 280 nm. This is because the − 100 V applied voltage is close to Geiger mode for device P1, and the avalanche gain is more likely to occur than that for other devices.

**Fig. 11:**
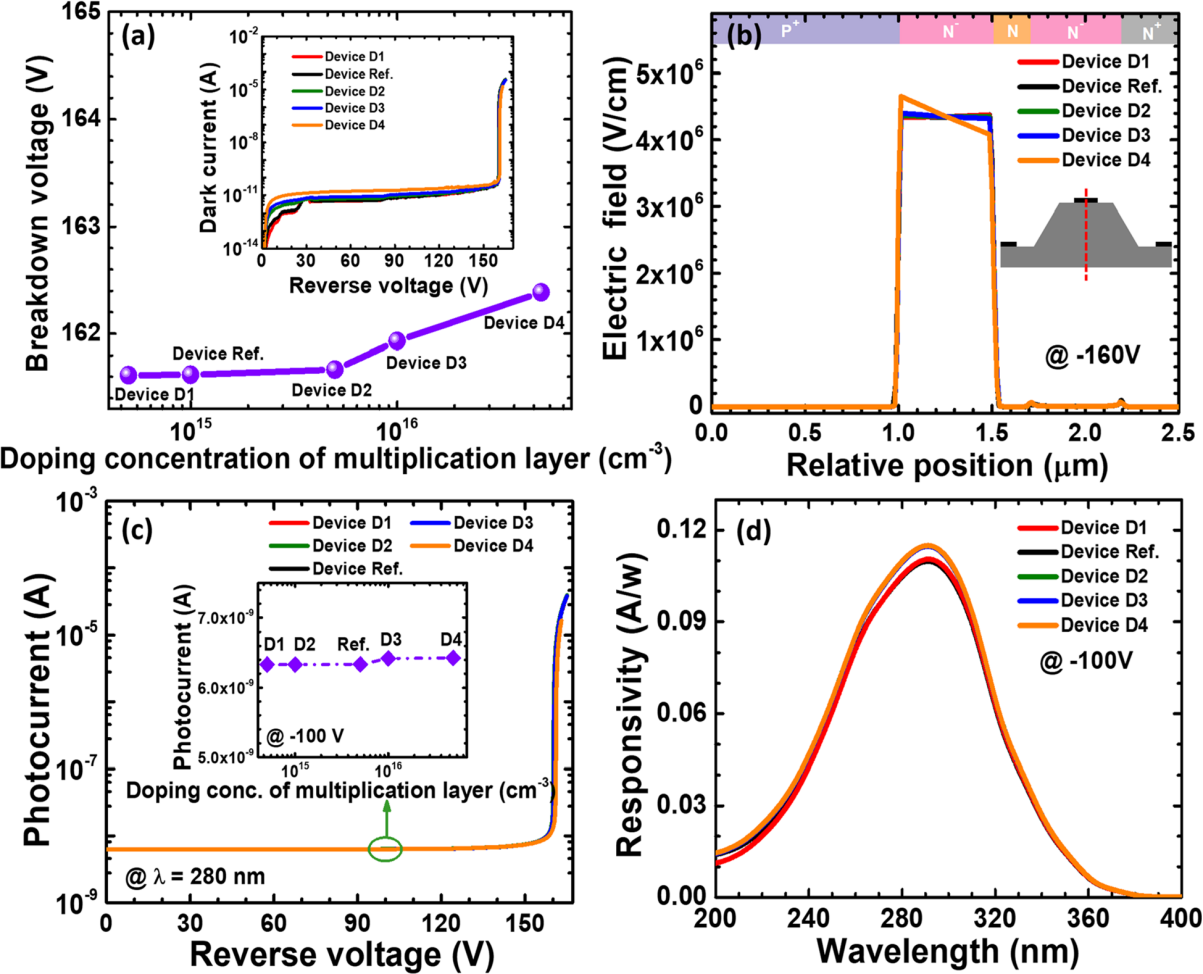
**a** Breakdown voltage, **b** vertical electric field distribution of the SACM APDs biased at − 160 V, **c** photocurrent-voltage characteristics under 280 nm illumination, and **d** spectral response characteristics of the SACM APDs biased at − 100 V for reference device and devices D1 to D4 with different doping concentration of multiplication layer, respectively. Inset figure in **a** shows the calculated current-voltage characteristics for reference device and devices D1 to D4. Inset figure in **c** shows photocurrent for reference device and devices D1 to D4 biased at − 100 V

Then, we show the breakdown voltage in terms of the multiplication layer doping concentration for reference device and devices D1 to D4 in Fig. 11a. It seems that when the doping concentration for the multiplication layer is lower than 10^16^ cm^−3^, the breakdown voltage is less affected. We believe the breakdown voltage can be significantly decreased if the doping concentration in the multiplication layer exceeds 10^18^ cm^−3^. The dark current as a function of the applied bias for the five APDs are shown in the inset of Fig. 11a. The dark current increases with increasing doping concentration of the multiplication layer due to the enhanced space charge generation in the depletion region. Therefore, for the purpose of significantly decreasing the dark current and promoting the carrier multiplication process, we rarely have the multiplication layer heavily doped. Then, we calculate the vertical one-dimensional electric field profiles for the five studied devices, which are demonstrated in Fig. 11b. We can see that the electric field profiles of the five devices are mainly confined in the multiplication layer. In addition, Figs. 11c and d demonstrate the photo-generated current and the wavelength-dependent responsivity for the five devices. We can see that the photo-generated current for reference device and devices D1 and D2 are almost the same under the 280 nm illumination, while that the photon-generated current for the devices D3 and D4 is slightly increased. Therefore, the responsivity at the wavelength of 280 nm in Fig. 11d for devices D3 and D4 is slightly higher than the others.

### Impact of the Beveled Mesa Angle on the Photoelectric Performance

In order to eliminate premature breakdown and suppress leakage current that are caused by the junction termination, positive beveled mesas with a small inclination angle are usually adopted when fabricating 4H-SiC APDs [13–16, 18]. However, the angles of the positive beveled mesa adopted in previous reports are various. Thus, to get systematic insight into the influence of different mesa inclination angles on the electric field profiles for 4H-SiC SACM APDs, we design the devices that are shown in Table 5.

**Table 5:** Structural parameters for the beveled mesa angle for the investigated devices

Angle for beveled mesa			
Device number	Angle	Device number	Angle
Device E1	6°	Device E3	20°
Reference device	8°	Device E4	40°
Device E2	10°	Device E5	90°

**Fig. 12:**
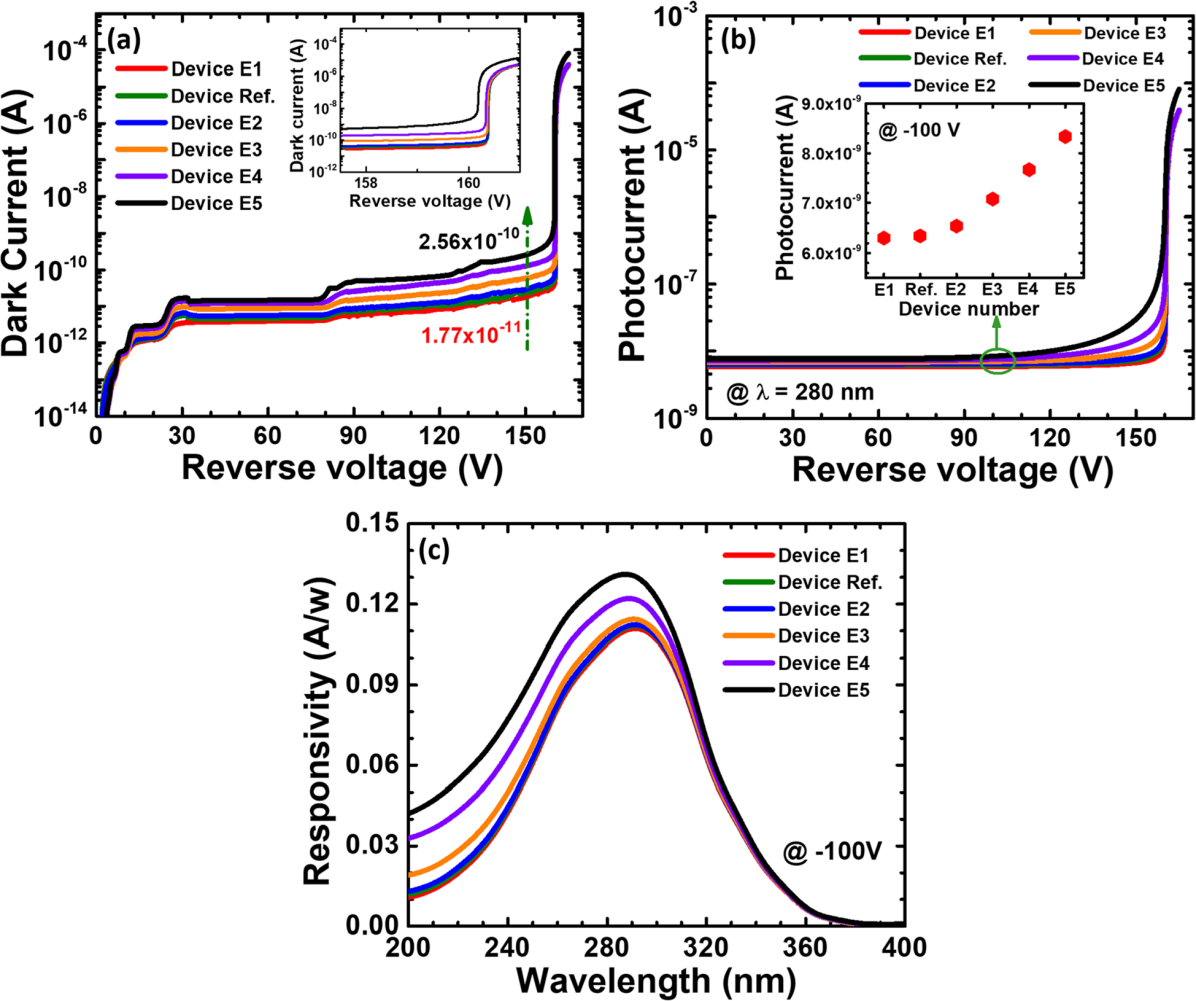
**a** Numerically calculated dark current-voltage characteristics, **b** photocurrent-voltage characteristics under 280 nm illumination, and **c** spectral response characteristics of the SACM APDs biased at − 100 V for reference device and devices E1 to E5, respectively

We firstly calculate and show the dark current-voltage characteristics for the six investigated devices with the various bevel angles in Fig. 12a. We can see that the dark current increases as the positive beveled angle becomes large [see Fig. 12a]. The breakdown voltages for the investigated devices are ~ 161.6 V except that device E5 is slightly less than 161.6 V. The premature breakdown is observed as the beveled mesa angle increase in the dark condition. Meanwhile, we calculate and show photo-generated current in terms of the applied bias for reference device and devices E1 to E5 in Fig. 12b. We also see that the photo-generated current also increases as the positive bevel increases according to the inset for Fig. 12b. The premature breakdown is also observed as the beveled mesa angle increase in Fig. 12b. Therefore, the responsivity of solar-blind waveband at − 100 V slightly enhances as the positive bevel angle increases according to Fig. 12c.

**Fig. 13:**
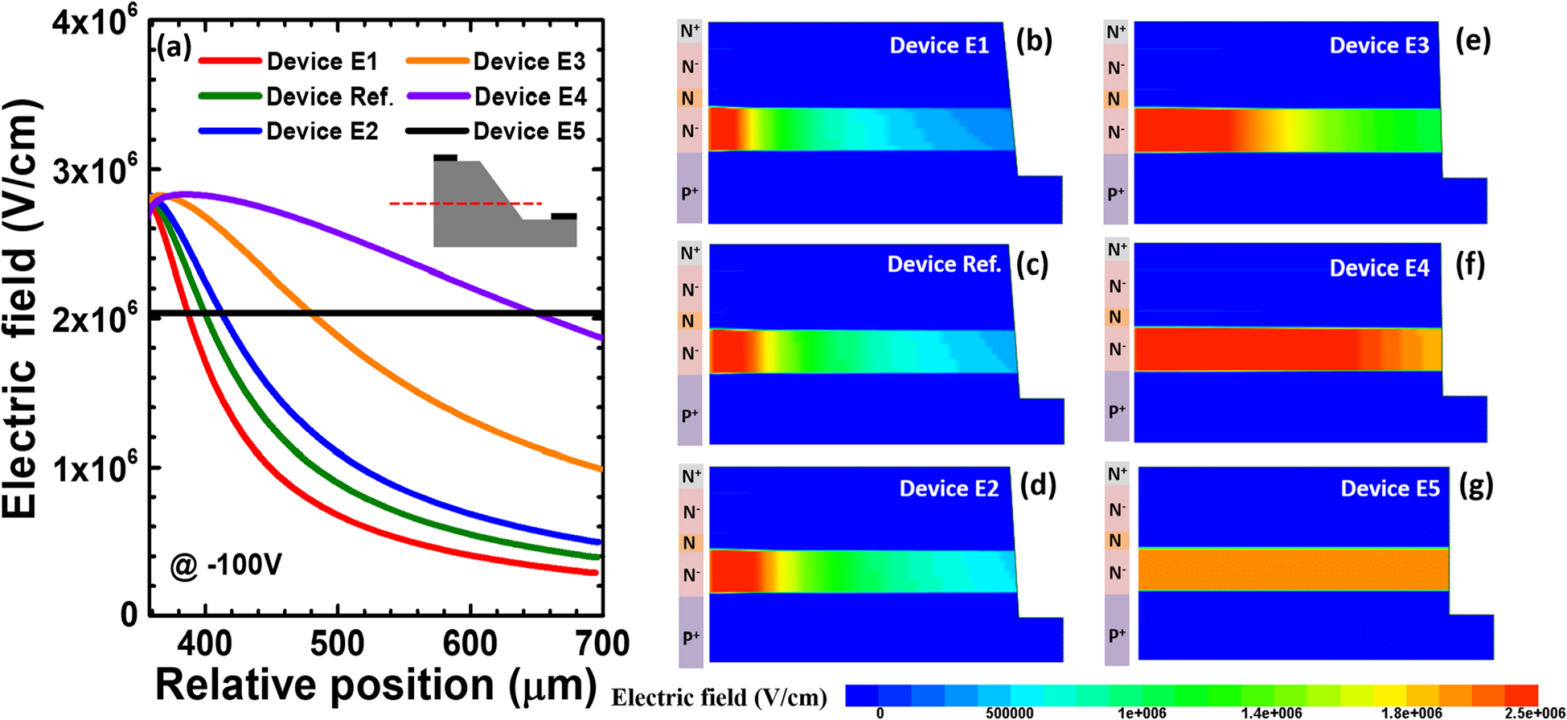
**a** Lateral electric field distribution of the multiplication layer at − 100 V, and numerically calculated two-dimensional electric field distribution at − 100 V for **b** device E1, **c** reference device, **d** device E2, **e** device E3, **f** device E4 and **g** device E5

To reveal the origin for the observations in Figs. 12a and b, we calculate the lateral electric field distribution in the multiplication layer at the reverse bias of − 100 V in Fig. 13a, which demonstrates that, when the beveled mesas are utilized, the electric field decreases from the mesa center to the mesa edge. Moreover, the edge electric field intensity drops as the angle further decreases for the investigated devices. As has been mentioned, the junction termination will cause a large number of surface imperfections, which may cause the premature breakdown and the strongly leakage current, and the adopting of the beveled mesa shifts the premature breakdown from the mesa surface to the bulk [29]. Moreover, to get a full picture for the electric field profiles, the two-dimensional electric field distributions at the reverse bias of − 100 V for reference device and devices E1 to E5 are presented in Figs. 13b-g. We can see that the area of the high electric field in the entire multiplication layer gets narrowed, and this simultaneously causes the carriers that regenerated by impact ionization to decrease. As can be seen from Table 6, as the beveled mesa angle decreases, the surface electric field at the relative position of 700 μm decreases from 2.03 × 10^6^ V/cm to 2.90 × 10^5^ V/cm. As a result, the surface leakage and bulk leakage can be further suppressed as the beveled mesa angle get further decreased as shown in Fig. 12a. Although a small beveled mesa angle is preferred, this sacrifices the active detection area for APDs, and therefore, the responsivity is the lowest for device E1 according to Fig. 12c. Thus, one shall properly optimize beveled mesa angles depending on the crystalline quality for the 4H-SiC epitaxial layers and the surface conditions after junction termination. The suggested beveled angle in this works is in the range of 10–20°.

**Table 6:** Surface electric field for the investigated devices

Device number	Device E1	Reference device	Device E2	Device E3	Device E4	Device E5
Surface electric field (V/cm)	2.90 × 10^5^	3.94 × 10^5^	4.96 × 10^5^	9.89 × 10^5^	1.86 × 10^6^	2.03 × 10^6^

## Conclusions

To summarize, we have numerically investigated and demonstrated the impact of the thickness and doping concentration of each layer on photoelectric performance for 4H-SiC SACM APDs. The obtained conclusions are as follows: (1) for n-type ohmic contact layer with a properly high doping concentration (*N*_*d*_ ≈ 1 × 10^19^ cm^−3^) to enable ohmic contact, the thickness and doping concentration hardly affect the breakdown voltage. Nevertheless, the responsivity decreases as the thickness of the n-type ohmic contact layer increases. The thickness shall be controlled to about 0.2 μm; (2) the doping concentration for the absorption layer is vitally important, which can modulate the photo-generated carrier transport and affect the responsivity. The doping concentration is generally controlled at the intrinsic concentration (*N*_*d*_ ≈ 1 × 10^15^ cm^−3^); (3) the doping concentration for the charge control layer regulates the electric field distribution and affects the depletion region width for 4H-SiC SACM APDs. The depletion region width increases as the doping concentration of the charge control layer decreases. According to our results, when the doping concentration is about 1 × 10^18^ cm^−3^, the depletion region can be completely terminated by the charge control layer; (4) the breakdown voltage can be strongly affected by the thickness of multiplication layer which is the main support region of the electric field. The dark current is sensitive to the doping concentration of multiplication layer, and a low doping concentration for the multiplication layer is required, since the doping concentration therein influences the space charge generation current. Thus, the suggested doping concentration in this works is intrinsic concentration (*N*_*d*_ ≈ 1 × 10^15^ cm^−3^); (5) we also point out the advantage of beveled mesa for 4H-SiC SACM APDs, and the optimized beveled mesa angles shall be a compromise among the active detection area, the surface conditions for the mesa, and the crystalline quality for 4H-SiC epitaxial films. This work indicates that the optimum beveled mesa angle is in the range of 10–20°. We strongly believe that this work provides the physical insight for the device physics and hence the findings in this work are very important for 4H-SiC-based SACM APDs.

## Data Availability

The data and the analysis in the current work are available from the corresponding authors on reasonable request.

## Abbreviations

AlGaN: Aluminum gallium nitride
APSYS: Advanced Physical Models of Semiconductor Devices
MSM: Metal-semiconductor-metal
SACM: Separated absorption charge and multiplication
SiC: Silicon carbide
SRH: Shockley-Read-Hall
UV APD: Avalanche ultraviolet photodiode

## References

[1] Prasai D, John W, Weixelbaum L, Krüger O, Wagner G, Sperfeld P, Nowy S, Friedrich D, Winter S, Weiss T (2013). Highly reliable silicon carbide photodiodes for visible-blind ultraviolet detector applications. J Mater Res.

[2] Alaie Z, Mohammad Nejad S, Yousefi MH (2015). Recent advances in ultraviolet photodetectors. Mat Sci Semicon Proc.

[3] Yang S, Zhou D, Cai X, Xu W, Lu H, Chen D, Ren F, Zhang R, Zheng Y, Wang R (2017). Analysis of dark count mechanisms of 4H-SiC ultraviolet avalanche photodiodes working in Geiger mode. IEEE T Electron Dev.

[4] Cai Q, Dong K, Xie Z, Tang Y, Xue J, Chen D (2019). Enhanced front-illuminated p-i-p-i-n GaN/AlGaN ultraviolet avalanche photodiodes. Mat Sci Semicon Proc.

[5] Chen B, Yang Y-T, Chai C-C, Zhang X-J (2011). Quantitatively exploring the effect of a triangular electrode on performance enhancement in a 4H-SiC metal-semiconductor-metal ultraviolet photodetector. Chin Phys Lett.

[6] Sciuto A, Roccaforte F, Di Franco S, Raineri V, Billotta S, Bonanno G (2007). Photocurrent gain in 4H-SiC interdigit Schottky UV detectors with a thermally grown oxide layer. Appl Phys Lett.

[7] Yang Sen, Zhou Dong, Lu Hai, Chen Dunjun, Ren Fangfang, Zhang Rong, Zheng Youdou (2016). 4H-SiC p-i-n Ultraviolet Avalanche Photodiodes Obtained by Al Implantation. IEEE Photonics Technology Letters.

[8] Li L, Zhou D, Lu H, Liu W, Mo X, Ren F, Chen D, Wang R, Li G, Zhang R, Zheng Y (2017). 4H-SiC avalanche photodiode linear array operating in Geiger mode. IEEE Photonics J.

[9] Sciuto A, Mazzillo M, Di Franco S, Roccaforte F, D'Arrigo G (2015). Visible blind 4H-SiC P^+^-N UV photodiode obtained by Al implantation. IEEE Photonics J.

[10] Razeghi M (2002). Short-wavelength solar-blind detectors-status, prospects, and markets. P IEEE.

[11] Liu T, Wang P, Zhang H (2015). Performance analysis of non-line-of-sight ultraviolet communication through turbulence channel. Chin Opt Lett.

[12] Shaw GA, Siegel AM, Model J, Geboff A (2009). Deep UV photon-counting detectors and applications. Proc of SPIE.

[13] Cai X, Zhou D, Yang S, Lu H, Chen D, Ren F, Zhang R, Zheng Y (2016). 4H-SiC SACM avalanche photodiode with low breakdown voltage and high UV detection efficiency. IEEE Photonics J.

[14] Yang S, Zhou D, Xu W, Cai X, Lu H, Chen D, Ren F, Zhang R, Zheng Y (2017). 4H-SiC ultraviolet avalanche photodiodes with small gain slope and enhanced fill factor. IEEE Photonics J.

[15] H.-Y. Cha, S. Soloviev, G. Dunne, L. Rowland, S. Zelakiewicz, P. Waldrab, and P. Sandvik (2006) Comparison of 4H-SiC separate absorption and multiplication region avalanche photodiodes structures for UV detection. In Proc. IEEE Sensors, 14–17

[16] Guo X, Beck AL, Huang Z, Duan N, Campbell JC, Emerson D, Sumakeris JJ (2006). Performance of low-dark-current 4H-SiC avalanche photodiodes with thin multiplication layer. IEEE T Electron Dev.

[17] Xin X, Yan F, Koeth TW, Joseph C, Hu J, Wu J, Zhao JH (2005). Demonstration of 4H-SiC visible-blind EUV and UV detector with large detection area. Electron Lett.

[18] Zhou X, Han T, Lv Y, Li J, Lu W, Wang Y, Song X, Tan X, Liang S, Feng Z, Cai S (2018). Large-area 4H-SiC ultraviolet avalanche photodiodes based on variable-temperature reflow technique. IEEE Elect Device L.

[19] Guo J, Yang Y, Wu F, Sumakeris J, Leonard R, Goue O, Raghothamachar B, Dudley M (2016). Direct determination of burgers vectors of threading mixed dislocations in 4H-SiC grown by PVT method. J Electron Mater.

[20] Nakamura D, Gunjishima I, Fineberg J (2004). Ultrahigh-quality silicon carbide single crystals. Nature.

[21] Zhou X, Li J, Lu W, Wang Y, Song X, Yin S, Tan X, Lv Y, Guo H, Gu G, Feng Z (2018). Large-area 4H-SiC avalanche photodiodes with high gain and low dark current for visible-blind ultraviolet detection. Chin Opt Lett.

[22] Chong E, Koha Y, Leeb D-H, Baeb I-H, Kima J-S, Jeonga Y-S, Ryua J, Leec J-Y, Kangc M-J, Parka J-H, Choi K-K (2019). Effect of beveled mesa angle on the leakage performance of 4H-SiC avalanche photodiodes. Solid State Electron.

[23] Sung W, Jayant Baliga B, Huang AQ (2016). Area-efficient bevel-edge termination techniques for SiC high-voltage devices. IEEE T Electron Dev.

[24] Berenguier B, Palais O, Bertaina S, Ottaviani L, Lyoussi A (2016). Lifetime measurement in n-type 4H-SiC by mean of the microwave phase-shift. Mater Sci Forum.

[25] Ng BK, David JPR, Tozer RC, Rees GJ, Yan F, Zhao JH, Weiner M (2003). Nonlocal effects in thin 4H-SiC UV avalanche photodiodes. IEEE T Electron Dev.

[26] Chen X, Zhu L (2010). Research and analysis on absorption coefficient of 4H-SiC at ultraviolet wavelength. Chin J Spec Lab.

[27] Cha H-Y, Sandvik PM (2008). Electrical and optical modeling of 4H-SiC avalanche photodiodes. Jpn J Appl Phys.

[28] Zhang Z-H, Tian K, Chu C, Fang M, Zhang Y, Bi W, Kuo H-C (2018). Establishment of the relationship between the electron energy and the electron injection for AlGaN based ultraviolet light-emitting diodes. Opt Express.

[29] Jayant Baliga B (2008). Fundamentals of power semiconductor devices.

